# Web-Based Apps for Reflection: A Longitudinal Study With Hospital Staff

**DOI:** 10.2196/jmir.3040

**Published:** 2014-03-17

**Authors:** Bettina Renner, Joachim Kimmerle, Dominik Cavael, Volker Ziegler, Lisa Reinmann, Ulrike Cress

**Affiliations:** ^1^Knowledge Media Research Center (KMRC) TuebingenGermany; ^2^Neurological Clinic Bad NeustadtBad NeustadtGermany; ^3^University of TuebingenTuebingenGermany

**Keywords:** computer applications software, mobile applications, learning, education, continuing, job satisfaction, hospitals, longitudinal studies, self report

## Abstract

**Background:**

Reflection is an important cognitive process in workplace learning; however, it occurs only rarely on its own and therefore needs additional support.

**Objective:**

In this study, we investigated the effect of software applications (apps) that aim to support reflection on hospital staff’s actual reflection behavior. In doing so, we also analyzed the relationship between reflection and the job satisfaction of health care professionals.

**Methods:**

Reflective learning was introduced in the ward of a neurological hospital by providing apps that aimed to foster particular aspects of individual and collaborative reflection. Data were collected repeatedly: once before the introduction of the apps and again 2 years after the initial measure. We used a questionnaire with subjective ratings of reflection and job satisfaction. Response rates were 34.4% (167/485) for the first and 40.6% (210/517) for the second measure.

**Results:**

Collaborative reflection was increased (*P*=.047) after the provision of the apps (2010: mean 2.84, SD 0.72; 2012: mean 3.06, SD 0.63) in contrast to a control group of other wards of the same hospital (2010: mean 2.68, SD 0.67; 2012: mean 2.63, SD 0.68). In addition, we revealed a positive correlation between collaborative reflection and job satisfaction (*r*=.61, *P*<.001).

**Conclusions:**

The findings provide evidence for an effect of the apps on hospital employees’ reflection behavior. Apps that foster reflective learning can increase health care professionals’ reflection about work experiences and support them in discussing experiences in teams or with their supervisors. The relationship between collaborative reflection and job satisfaction suggests that opportunities for joint reflection on work experiences in a hospital have further impact over and above fostering reflective learning per se. We discuss the limitations of our study and provide suggestions for both future research and the development of Web-based apps.

##  Introduction

### Background

Learning and the continuing development of employees are important issues in hospitals. Confronted with complex problems and stressful situations, nurses and physicians always need to keep their skills up to date and develop them further [1-3]. This happens not only through formal training but also informally in the daily routine of health care professionals [4-6]. Hospital management needs not only skilled and competent employees but also contented employees, since dissatisfied nurses and physicians are more likely to resign [7,8]. Job satisfaction of health care staff is related to patient satisfaction [8] and patient care (for an overview see [7]) and therefore plays an important role when it comes to the quality of medical care.

The aim of the present study is to examine an easy and efficient way of supporting reflective learning in hospital staff. For this purpose, we tested the suitability of application software (apps) that support reflection. Our aim was also to examine the relationship between reflection and job satisfaction of hospital staff.

We present theoretical and empirical considerations of reflection processes in the workplace and of job satisfaction, with each presentation leading to a concrete hypothesis. Then we describe the methods of this longitudinal study and its results. Concluding, we discuss our findings with respect to their practical implications and suggestions for future research in medical informatics.

### Reflection

One informal way of learning at work is through reflecting on work situations that employees themselves or colleagues have experienced [9,10]. Reflection is a process “in which people recapture their experience, think about it, mull it over and evaluate it” [11]. Reflection is considered to be an important process in organizations, for example, for innovation [12] or for organizational learning [13,14]. Although reflection is quite a natural process [9], people often do not use it consciously or intentionally as a skill to improve learning and performance [15]. Moreover, there are several other preconditions for the successful application of reflection in the workplace. On the part of the individual, not only a lack of awareness of the benefits of reflection, but also an absence of motivation for reflective thinking, as well as a lack of metacognitive skills like self-monitoring and self-evaluating might hinder reflective learning [16]. On the part of the organization, there are also certain conditions necessary to foster reflective thinking. An organization that wants employees to engage in reflection needs to give them opportunities to participate in shaping their work environment, at least in some areas. If an organization is characterized by strong hierarchies and employees are not involved in decision-making processes, the potential for change through reflective thinking is quite limited [16]. In sum, reflection, whether carried out individually or in teams, does not tend to occur by itself [12], rather, it needs support [13].

One area where reflection and reflective thinking are broadly researched topics is in the health care sector [3,17-20]. A special focus of previous research has been on the education of nurses [17-21] and physicians [1,22-26]. In hospitals, reflection helps the staff combine theory and practice [3,20] and enables them to turn experiences into learning opportunities [27]. In this way, reflection and reflective work behavior foster continuing professional development [28]. Research findings in this field suggest, however, that reflectivity is indeed not an easily applied skill. Instead, guidance and support are needed to achieve higher levels of reflection [21].

Since reflection is rare, but important and valuable for health care professionals, research needs to find approaches that can support this process [23]. One option that is relevant from a medical informatics perspective is the development of Web-based or mobile apps [29-31] to assist individuals and teams in their reflection processes in hospitals. These apps are supposed to support staff in exercising different aspects of reflection. Apps can remind people to reflect on their experiences and therefore compensate for low awareness or motivation. They can also extend the range of situations and experiences a single person faces, for example, by making additional experiences possible through virtual worlds or computer simulations. Moreover, apps can support collaborative reflection. Through collaborative reflection, people can learn from others and benefit from their experiences [32]. Since memories of experiences can become imprecise over time, it is important to capture them in such a way that they can be used for further reflection [33]. Apps can foster reflection in different ways, by providing experiences (eg, in virtual settings), documenting experiences, triggering reflection about experiences, and supporting communication and cooperation in collaborative reflection. So the first hypothesis of the current study is that (1) apps designed to foster reflection of health care professionals have a positive impact on the reflection activity of hospital staff.

### Job Satisfaction

One reason why people learn for their job is that the tasks of the job require it. But this is not the only motive. People also learn because it helps them in their career and because they have certain personal goals [6]. These goals comprise, among others, a need for personal achievement and development, and a feeling of autonomy [6]. Job satisfaction can be interpreted as the sum of the job expectations employees have that are met [34]. This means not only monetary expectations but also the wish to reach personal goals of development and achievement.

Consequently, we expect that job satisfaction is increased by good learning conditions. Some empirical evidence already exists for the potential influence of learning on job satisfaction. In different studies, significant correlations between organizational learning and job satisfaction [35], as well as between workplace learning and job satisfaction [36-38] have been found. Other authors have identified organizational learning culture as a predictor for general job satisfaction [39,40]. There is also support for the assumption of a relationship between learning and job satisfaction in the health sector. A strong correlation has been identified between professional growth and job satisfaction among nurse practitioners [41].

Because reflective learning is one type of informal learning that enables employees to develop and shape their working environment to a certain degree, we hypothesize that fostering reflection leads to an increase in job satisfaction. So the second hypothesis to be tested in this study is that (2) reflection is positively related to the job satisfaction of hospital staff.

## Methods

### Study Design and Setting

A longitudinal study was designed with the staff of a neurological hospital as participants. The study represented a 2 × 2 factorial design with reflection apps and time of measurement as between-subject factors. Due to privacy protection regulations, it was not possible to connect the data of the two measurement points on an individual level. Therefore, time of measurement could not be treated as a within-subject factor. Accordingly, we cannot completely rule out that the samples at the two points of measurement included slightly different individuals. But there was a low turnover rate in this hospital (around 3%); during the study, only two members left the unit that served as the experimental group. Reflection and job satisfaction were measured both before (2010) and after the introduction of the apps (2012).

The staff in one ward of the hospital participated as the experimental group. Participants in this group had the opportunity to use the apps. The staff in all the other wards of the hospital served as the control group. Participants in this group were not acquainted with the apps. The experimental group were ward personnel caring for patients suffering from acute strokes and other neurological emergencies (“Stroke Unit”). The staff of this ward consisted mainly of nurses and physicians. Participants of the control group were staff either of a ward treating all different kinds of neurological injuries, of a neurological intensive care unit, of a rehabilitation unit, or interdivisional staff.

### Procedure and Material

In the period between the two data collections, participants were involved in user studies and workshops in order to examine the reflection behavior and needs of hospital staff. Those findings were accounted for in the development of the apps. After the development stage, participants in the experimental group tested the apps in workshops and used them during their everyday work. The developing and testing phases together covered the entire time span between the two points of measurement. During this time, several 1-day workshops as well as two user studies were conducted in order to give the entire staff the chance to participate. The participants were provided with devices to make sure everyone could use the apps and nobody was prevented from participation. The apps addressed the different aspects of reflection as described above.

DocTrain is an app for mobile phones as well as a Web application. It was developed to support physicians during their specialist training. It reminds its users to reflect upon their experiences and helps them document their tasks and capture and share with their mentor their progress in training elements. The app provides a list of tasks, and the users can connect them via the barcode of a patient case with a particular patient. Users can also add personal notes. Data can then be used to reflect about a case individually or with a mentor [42].

CLinIC-The Virtual Tutor is a serious game. It was created to help nursing staff and physicians reflect on and prepare for difficult situations with patients. With the help of the game, users are provided with virtual experiences in handling difficult conversations with patients in order to be better prepared for corresponding situations in real life. A virtual tutor provides feedback and advice. Users can also take notes to reflect on them later. The set of tasks in the game is complemented by so-called mini games. These mini games are integrated into the branching stories of the game as a whole. They allow users to practice skills that are required in the health sector, such as recognizing emotional facial expressions and reflecting on them [43,44].

Finally, the Talk Reflection App allows users to reflect collaboratively on difficult situations with patients and relatives [45]. It consists of a forum where users can contribute experiences with difficult conversations and reflect on them asynchronously together with colleagues. Users can document problematic experiences, share them with others, and add their own thoughts about the situations privately or publicly. Others can then contribute to this topic. In addition, users can document their reflection outcomes [46]. So the app facilitates reflection by capturing data and provides support for coordination and communication in collaborative reflection.

### Data Collection

A survey with the hospital staff was conducted twice during the study. The first data collection took place in November and December 2010. The second measurement point was October/November 2012. The response rate was 34.4% in 2010 and 40.6% in 2012.

### Participants

In 2010, 167 employees of the hospital participated in the survey: 21 individuals worked in the Stroke Unit, 132 individuals in other wards of the hospital, and 14 did not indicate their ward affiliation. In 2012, 210 employees answered the questionnaire: 17 members of the Stroke Unit, 164 staff members of other wards, and 29 participants did not indicate their ward. The difference in group sizes between the experimental and the control group is due to the fact that the participants of the Stroke Unit were compared to the staff of all the other wards of the hospital; however, response rates did not differ between conditions (all *P*s>.05). Staff consisted of nurses, different kinds of therapists, physicians, and administration staff. Participants not indicating their ward were excluded from further analyses, so 334 individuals were considered for the investigation.

### Instruments

A 10-item questionnaire was used to measure reflection. Items were created during the research project MIRROR [47] on the basis of those aspects of reflection specified in the literature that were relevant to this study. Particularly relevant are the needs to reflect and to develop through reflection [48]. These aspects concern the motivational side of reflection, since motivation is considered an important precondition for the occurrence of reflection [10]. Another important requirement is the absence of barriers such as time pressure [4,10]. The positive consequences of collaborative reflection in terms of sharing and discussing experiences and different perspectives are described by several authors (eg, [4,9,10,13,49]). Collaborative reflection includes team reasoning, that is, the collective analysis of problems [9,12,50]. Collaborative reflection also includes support by a person with greater experience, such as a mentor or a supervisor [9]. To make sure that the items were comprehensible to the participants, their content was discussed with various hospital representatives beforehand, and some revisions were made [47]. The items that resulted from this procedure are listed in Table 1. Participants rated these items on 4-point Likert scales from 1=totally disagree to 4=totally agree.

In addition, participants were also asked 8 questions regarding general job satisfaction (Table 2). Participants rated these items also on 4-point Likert scales from 1=totally disagree to 4=totally agree. Moreover, they were asked to indicate their occupation group and the ward they worked in.

**Table 1:** Reflection items.

No.	Item
1	I often have the need to think about my work experiences.
2	I am provided with enough time to reflect upon my experiences at work.
3	It helps me for my development to think about my work experiences.
4	We discuss on a regular basis if we work successfully together as a team.
5	If things do not work out as they should, we try as a team to find the reason.
6	We as a team think about how we can learn from past experiences.
7	When I think I did not do a good job, I discuss with colleagues how I could improve.
8	When I think I did not do a good job, I discuss with my supervisor how I could improve.
9	I often think about how we can improve things in our organization.
10	In our organization we are encouraged to reflect upon our experiences at work.

**Table 2:** Job satisfaction items.

No.	Item
	In general, I am satisfied with...
1	…my work situation.
2	…the advanced and professional training.
3	…the work climate.
4	…the communication.
5	…the operational procedures.
6	…the quality of care.
7	…the leadership of my supervisor.
8	…the neurological hospital as employer.

### Statistical Analysis

To assess the internal consistencies of the scales, we calculated Cronbach alpha values and conducted an exploratory factor analysis for the reflection scale. For testing hypothesis 1, we conducted a contrast analysis [51]. The second time of measurement (2012) in the Stroke Unit ward was tested against the three other conditions (Stroke Unit 2010; other wards 2010 and 2012). For conducting the contrast analysis, we treated the 2 (2010 vs 2012) × 2 (Stroke Unit vs other wards) factors as a 1 × 4 design. The hypothesis that there was a higher level of reflection in the Stroke Unit in 2012 than in all other conditions (A>B=C=D) is represented in the focal contrast with the coefficients 3 -1 -1 -1. We also conducted an analysis of single items using *t* tests. For testing hypothesis 2, we calculated Pearson product-moment correlations for both the complete scales and for single items. Statistical analyses were conducted using IBM SPSS Statistics 20.

### Ethical Considerations

Data collection was approved by the hospital’s works council and the data protection officer. Participants signed a consent form that was also approved by the works council and the data protection officer.

## Results

### Internal Consistency and Factor Analysis

The complete reflection scale had an internal consistency of alpha=.84. To further explore the structure of the scale, a factor analysis was conducted extracting two factors, using a Varimax rotation. The two factors explained 56% of the variance that emerged (factor 1: 37%, factor 2: 19%). After rotation, all items had factor loadings of at least .50 on one of the two factors and not more than .40 on the respective other factor. The first factor was labeled *collaborative reflection* and the second factor labeled *individual reflection*. The collaborative reflection subscale consisted of 7 items (items 2, 4-8, and 10 in Table 1) and had an internal consistency of alpha=.85. The corrected item-total correlations of these items are presented in Table 3. The individual reflection subscale consisted of 3 items (items 1, 3, and 9 in Table 1) and had only an internal consistency of alpha=.59. Therefore, we had to exclude this subscale from further analyses. The internal consistency of the job satisfaction scale was alpha=.89.

**Table 3:** Corrected item-total correlations of the collaborative reflection items.

Item	Corrected item-total correlations
I am provided with enough time to reflect upon my experiences at work.	.38
We discuss on a regular basis if we work successfully together as a team.	.66
If things do not work out as they should, we try as a team to find the reason.	.68
We as a team think about how we can learn from past experiences.	.75
When I think I did not do a good job, I discuss with colleagues how I could improve myself.	.59
When I think I did not do a good job, I discuss with my supervisor how I could improve myself.	.64
In our organization we are encouraged to reflect upon our experiences at work.	.62

### Reflection

To test whether there was more reflection in the Stroke Unit in 2012 compared to 2010 and to the other wards (hypothesis 1), we conducted a contrast analysis with collaborative reflection as a dependent variable. To check whether there was systematic variance besides the variance of the focal contrast, we computed additional orthogonal contrasts. If the hypothesis of the focal contrast was correct, only the focal contrast but not the residual contrasts should be significant.

Regarding collaborative reflection, the hypothesis was supported by the data. There was a significant effect of the focal contrast: *F*_(1, 327)_=3.97, *P*=.047, eta squared=.01. But there was no effect of the associated orthogonal effects: *F*_(1, 327)_=1.90, *P*=.17. So the contrast analysis shows that collaborative reflection increased in the Stroke Unit from 2010 (mean 2.84, SD 0.72) to 2012 (mean 3.06, SD 0.63) but not in the other wards (2010: mean 2.68, SD 0.67; 2012: mean 2.63, SD 0.68).

In order to provide more information regarding their construct validity [52], we also considered the particular items of the scale and compared the data of the Stroke Unit in 2012 with the combined other conditions on item level. Results are presented in Table 4. For five of the seven collaborative reflection items, we found significantly higher means for the Stroke Unit in 2012 compared to the other conditions.

**Table 4:** Collaborative reflection items: means, standard deviations, *t*, and *P* values.

Item	Stroke Unit 2012, mean (SD)	Other conditions, mean (SD)	*t* (df)	*P* (one-tailed)
I am provided with enough time to reflect upon my experiences at work.	2.81 (0.91)	2.38 (0.79)	2.14 (323)	.02
We discuss on a regular basis if we work successfully together as a team.	3.35 (0.70)	2.53 (0.99)	4.59 (19.65)	<.001
If things do not work out as they should, we try as a team to find the reason.	3.41 (0.71)	2.96 (0.93)	2.00 (328)	.02
We as a team think about how we can learn from past experiences.	3.35 (0.61)	2.90 (0.91)	2.00 (329)	.02
When I think I did not do a good job, I discuss with colleagues how I could improve myself.	3.00 (0.87)	2.98 (0.93)	0.08 (326)	.47
When I think I did not do a good job, I discuss with my supervisor how I could improve myself.	2.88 (1.17)	2.37 (1.03)	1.98 (326)	.03
In our organization we are encouraged to reflect upon our experiences at work.	2.65 (1.00)	2.50 (0.91)	0.67 (322)	.25

### Job Satisfaction

In order to test hypothesis 2, we computed a Pearson product-moment correlation to analyze the relationship between collaborative reflection and job satisfaction measurements. As expected, the collaborative reflection scale was positively correlated to the job satisfaction scale (*r*=.61, *P*<.001).

Analogous to the presentation of the reflection results, we considered the single items of the collaborative reflection scale in order to provide more information regarding their construct validity. For this purpose, we computed Pearson product-moment correlations for job satisfaction with the individual items of the collaborative reflection scale. As shown in Table 5, all items of the collaborative reflection scale were positively correlated to job satisfaction (all *P*s<.001).

**Table 5:** Significant correlations of the collaborative reflection items with job satisfaction.

Item	*r*
I am provided with enough time to reflect upon my experiences at work.	.43
We discuss on a regular basis if we work successfully together as a team.	.38
If things do not work out as they should, we try as a team to find the reason.	.49
We as a team think about how we can learn from past experiences.	.45
When I think I did not do a good job, I discuss with colleagues how I could improve.	.37
When I think I did not do a good job, I discuss with my supervisor how I could improve.	.42
In our organization we are encouraged to reflect upon our experiences at work.	.58

## Discussion

### Main Findings

The first goal of this study was to test whether the introduction of apps that support reflection would affect hospital staff’s actual reflection behavior at the workplace. We found an increase in collaborative reflection for those hospital employees who had the opportunity to use the apps. With regard to the contents of the reflection items, our findings illustrate which aspects of reflection the apps were particularly able to support. The apps facilitated the conjoint aspects of reflection. In 2012, participants in the Stroke Unit indicated to a higher degree that they discuss on a regular basis their work as a team. They also stated an increased effort to collaboratively learn from past experiences and to find reasons if something did not work out as it was intended. App users also indicated they increased communication with their supervisors. These are aspects that were explicitly addressed by the apps, since they aimed directly at collaborative reflection. The Talk Reflection app, for instance, was designed with the ambition of fostering collaborative reflection. DocTrain was not only designed for individual reflection but also for reflecting with a mentor. Finally, CLinIC simulated colleagues in the game and allowed for collaborative reflection as an opportunity to improve performance.

As expected, there was a positive relationship between reflection behavior and job satisfaction, indicating that higher levels of collaborative reflection processes go along with enhanced job satisfaction among participants in the hospital. This further supports the findings of other authors regarding a relationship between informal learning and job satisfaction as introduced above. The content of our collaborative reflection scale suggests that being provided with sufficient time to reflect, reflecting collaboratively in a team, and discussing work with a supervisor go along with a higher level of job satisfaction.

### Limitations and Future Work

A limitation of this study is that there were three different reflection apps. Thus, it is not possible to say whether it was the co-actions of all the apps or only a particular app with certain features that led to the increase in reflection. The DocTrain app, for instance, mainly captures data for reflection. CLinIC provides users with additional experiences in a virtual world where they can test different reactions to a situation without doing any harm. But even a single app might itself foster reflection in diverse ways. In the case of the Talk Reflection app, for example, it could be that just writing down their problems helps users to think about them in a different way. But it could also be that the comments of their colleagues help them to acquire new insights. Future studies with single specific reflection apps should be designed to shed light on these questions.

Another issue is the topic of collaborative reflection. All apps aimed at fostering collaborative reflection. And in fact, participants in the experimental condition indicated that there was more reflection in the team after the introduction of the apps. However, it remains unclear whether this increase in reflective activity was directly affected by the apps, or whether it just happened that people started to talk about the experiences they had using the apps, or a combination of both. Our data do not ultimately indicate how often and in which way participants used the apps during their everyday work. Future studies should be designed in a way that allows capturing pertinent data about usage. In addition, we did not entirely evaluate the extent to which each individual item of the collaborative reflection scale represented typical reflection behavior from the perspective of the participants. Further research using qualitative methods should assess the representativeness of these items with respect to collaborative reflection.

Another limitation of the study is the character of the analysis of the relationship between reflection and job satisfaction. As the relationship could only be shown here correlatively, future research should study this relationship in more detail in order to find out under which conditions reflection apps increase general job satisfaction. Finally, it might be worthwhile for future research to examine the connection between app-induced reflection and other socio-emotional experiences of health care professionals over and above job satisfaction.

### Conclusions

The current study shows that collaborative technology is relevant for health care professionals [53] and that apps that support reflection have an impact on the collaborative reflection behavior of hospital staff. App designers should therefore have a special eye on the issue of collaborative reflection. Developers should also keep in mind that apps might preferably be used by the whole team of a hospital unit. In order to accommodate this preference, developers should consider the different occupation groups in their app design in order to facilitate multidisciplinary collaboration. Reflection apps should provide opportunities for simple and self-evident interactions with colleagues.

We also found that collaborative reflection is related to the job satisfaction of hospital staff, further indicating the high relevance of reflection for health care professionals. This provides an additional reason for designers of medical apps to look into this topic. Reflection apps seem to have strong potential in various respects. They may not only facilitate users’ cognitive processes, such as improving their ways of learning and working. Apps that foster reflection also seem to have the potential to support health care professionals on a socio-emotional level.
